# “Liquid Biopsy” of White Matter Hyperintensity in Functionally Normal Elders

**DOI:** 10.3389/fnagi.2018.00343

**Published:** 2018-11-09

**Authors:** Fanny M. Elahi, Kaitlin B. Casaletto, Marie Altendahl, Adam M. Staffaroni, Evan Fletcher, Teresa J. Filshtein, Maria M. Glymour, Bruce L. Miller, Jason D. Hinman, Charles DeCarli, Edward J. Goetzl, Joel H. Kramer

**Affiliations:** ^1^Memory and Aging Center, Department of Neurology, University of California, San Francisco, San Francisco, CA, United States; ^2^Department of Neurology, University of California, Davis, Davis, CA, United States; ^3^Department of Epidemiology and Biostatistics, University of California, San Francisco, San Francisco, CA, United States; ^4^Department of Neurology, University of California, Los Angeles, Los Angeles, CA, United States; ^5^Department of Medicine, University of California, San Francisco, San Francisco, CA, United States; ^6^Jewish Home of San Francisco, San Francisco, CA, United States

**Keywords:** cerebral small vessel disease, exosomes, extracellular vesicles, biomarkers, white matter, inflammation

## Abstract

**Background and Objective:** In the aging brain, increased blood-brain barrier (BBB) leakage and white matter hyperintensity (WMH) on MRI are frequently presumed secondary to cerebral small vessel disease (cSVD) or endotheliopathy. We investigate this association *in vivo* by quantifying protein cargo from endothelial-derived exosomes (EDE), and comparing levels between two groups of functionally normal elders with and without WMH. In addition, we study associations of EDE proteins with upstream and downstream factors, such as inflammation and neurodegenerative changes, respectively.

**Methods:** Twenty six neurologically normal older adults completed general health questionnaires, neuropsychological and physical examinations, and brain MRI. WMH was visually graded with modified Fazekas score of 2 or greater used to classify 11 subjects as cases, and 15 without WMH as controls. Plasma total exosomes were precipitated and EDEs enriched by sequential immuno-precipitations. In addition, we quantified three inflammatory cytokines from plasma and imaging variables on MRI. Group means were compared, the discriminant functions of biomarkers calculated, and the association of EDE biomarkers with plasma inflammatory markers, cognition, and imaging outcomes assessed via regression modeling.

**Results:** Plasma levels of EDE cargo proteins GLUT1, LAT1, P-GP, and NOSTRIN were significantly higher in subjects with WMH in comparison to those without. In contrast, EDE levels of the marker with low expression in brain (VCAM1) were equal between groups. The effect sizes for each of the brain-expressed cargo proteins (GLUT1, LAT1, and P-GP) were such that age-adjusted logistic regressions revealed areas under the curve (AUC) with range of 0.82–0.89, differentiating subjects with WMH from those without. VCAM1 poorly discriminated between groups (AUC:0.55). Higher levels of all brain-expressed EDE proteins were also associated with lower cognitive function, unrelated to burden of WMH. Levels of LAT1 and P-GP were significantly inversely associated with global gray matter volumes, and EDE GLUT1, LAT-1, and P-GP concentrations were significantly associated with systemic IL-6 levels.

**Conclusion:** In a case control study of clinically normal adults with and without WMH, concentrations of EDE proteins were significantly higher in subjects with WMH in comparison to controls. This work is a first step toward *in vivo* dissection of molecular changes in endothelia of functionally normal subjects with radiographic evidence of age-associated white matter disease.

## Introduction

White matter hyperintensity (WMH) on brain MRI has been associated with slowed processing speed and cognitive decline (Fazekas et al., 1987; Filley and Fields, 2016). The pathological substrate of WMH can be heterogenous (Wardlaw et al., 2015), putatively resulting from the dysfunction of any of the cellular constituents of blood-brain barrier (BBB) and increased leakage at this interface. In the aging brain, when localized to periventricular or subcortical regions, and insidiously progressive, BBB leakage and WMH are frequently presumed secondary to cerebral small vessel endotheliopathy (Fazekas et al., 1987; Zhang et al., 2016; Li et al., 2017, 2018; Gupta et al., 2018). Consequently, endothelial disease and BBB dysfunction are emerging as major contributors to cognitive change in the elderly (Schneider et al., 2007; Schneider and Bennett, 2010; Attems and Jellinger, 2014). Several pathogenic mechanisms are hypothesized to underlie the noted cognitive decline, including metabolic changes, inflammation, CNS immune activation, and downstream astrogliosis and neurodegeneration (McAleese et al., 2016). At a time when targeted molecular therapies are being developed, a major challenge has been the low specificity of fluid and structural biomarkers for quantification of endothelial contributions to BBB dysfunction and neurodegeneration. Endothelial-derived exosomes (EDE) biomarkers allow direct interrogation of endothelial cells and the investigation of associations with risk factors and downstream pathological changes.

Exosomal molecular cargo have emerged as promising biomarkers of disease processes within all organs, including the brain (Fiandaca et al., 2015; Goetzl et al., 2015a,b, 2016; Mustapic et al., 2017; Zhao and Zlokovic, 2017). Exosomes are plasma membrane- and endosomal-derived vesicles that are released by most cell types and contain cargo molecules from their cell of origin including proteins, mRNAs, microRNA, and lipids (Piper and Katzmann, 2007; Kanninen et al., 2016). Cerebral cell-derived exosomes range in diameter from 30 to 220 nm and are capable of crossing the BBB (Chen et al., 2016). Analyses of exosome-derived molecules isolated from bodily fluids are providing an unprecedented ability to non-invasively investigate molecular changes in specific cells *in vivo*, with great impact on diagnostics of diseases affecting inaccessible organs (Armstrong and Wildman, 2018). As stated earlier, cerebral small vessel disease (cSVD) has thus far been quantified via downstream pathophysiological changes reflected in non-specific signal alterations on neuroimaging, such as WMH on T2 and fluid-attenuated inversion recovery (FLAIR) (Fazekas et al., 1987; Zeestraten et al., 2017). Differences in mean concentrations of specific EDE proteins have recently been demonstrated in individuals with known systemic and cerebral vascular disease (Goetzl et al., 2017). Little is known, however, about the relationship between the selected EDE proteins in the clinically silent phase of disease. In this proof-of-concept study we investigate EDE proteins in subjects with and without WMH on FLAIR imaging in order to evaluate their utility in early, asymptomatic disease phase. To this end, we selected EDE proteins predominantly expressed in brain endothelial cells and involved in metabolic control (GLUT1), BBB function (P-GP, LAT1), or regulation of perfusion and inflammation (NOSTRIN), as well as a protein involved in inflammatory vascular changes and leukocyte migration (VCAM1), but most notably expressed in systemic rather than brain vasculature.

To our knowledge, this is the first study that reports on EDE cargo protein differences in asymptomatic, clinically normal subjects with and without WMH on brain MRI. We additionally examined the association of EDE cargo protein concentrations with a major vascular risk factor, inflammation, and downstream cognitive and structural brain changes, such as slowed processing speed, and gray matter atrophy.

## Materials and Methods

### Study Participants

To minimize bias, we performed a consecutive sampling of functionally intact, older study participants from ongoing longitudinal studies of brain aging at the Memory and Aging Center at UCSF (NIH Aging and Cognition study; Larry L. Hillblom foundation study; NIH Chronic Inflammation study) to prospectively participate in this study. The inclusion criteria for all subjects comprised intact daily functioning per an informant (CDR = 0), neuropsychological performances within normative standards, and absence of significant clinical neurological disease assessed by history and physical exam. The study participants were selected based on evidence or absence of white matter injury on brain MRI. We selected 11 cSVD cases classified based on global cerebral volume of WMH on FLAIR/T2, corresponding to modified Fazekas score of 2 or greater. We selected 15 controls who had no significant WMH on brain MRI (Fazekas = 0), or other significant abnormalities, such as focal atrophy. All images were rated for burden of WMH by a board-certified neurologist (FME) in addition to being reviewed by a neuroradiologist to rule out of other significant abnormalities. At the time of enrollment in the current study, each participant completed a blood draw for preparation of a platelet-poor-plasma according to previously published protocol (Goetzl et al., 2017). Plasma samples were aliquoted and stored in 0.25 ml aliquots at −80°C until used for biochemical processing. All study participants were provided informed consent and the study protocols were approved by the UCSF Committee on Human Research. Research was performed in accordance with the Code of Ethics of the World Medical Association.

### Cognition (Processing Speed)

Participants completed a modified version of the Trail-Making Test, a measure of speeded set-shifting (Kramer et al., 2003), which is commonly affected by cSVD. This task requires participants to sequentially alternate between numbers and days of the week as quickly as possible. The outcome variable is the time taken to perform the task (log transformed to normalize the distribution).

### Neuroimaging Evaluation

#### MRI Acquisition

Subjects were scanned on a Siemens Prisma 3T scanner at the UCSF Neuroscience Imaging Center. A T1-weighted magnetization-prepared rapid gradient echo (MP-RAGE) structural scan was acquired in a sagittal orientation, a field-of-view of 256 × 240 × 160 mm with an isotropic voxel resolution of 1 mm^3^, TR = 2300 ms, TE = 2.9 ms, TI = 900 ms, flip angle = 9°. The T2-weighted FLAIR was acquired in the axial orientation, field-of-view = 176 × 256 × 256 mm, resolution 1.00 × 0.98 × 0.98 mm3, TR = 5000 ms, TE = 397 ms, TI = 1800 ms.

#### MRI Processing and Analyses

De-identified digital information was transferred from UCSF using secure and HIPAA compliant DICOM server technology. Images were processed by the IDEAS lab and full imaging protocol details are reported in prior publications (DeCarli et al., 2005a,b; Maillard et al., 2011, 2012). In brief, WMH volumes were obtained from T2-weighted FLAIR images using an automated method for quantification and localization of WMH. Total WMH volume was estimated by summing all the voxels classified as WMH and normalized for total intracranial volume.

### Enrichment of Plasma EDEs and Extraction of Cargo Proteins

Platelet-poor plasma was prepared from 6 ml of venous blood and stored in 0.25 ml aliquots at −80°C as previously described (Goetzl et al., 2017) and EDE were enriched as per previously published protocol (Goetzl et al., 2017). Briefly, after depletion of platelets, EDE exosomes were enriched sequentially by immunoprecipitation with two biotinylated monoclonal antibodies to CD31 (MEM-05, Thermo Fisher Scientific) and CD146 (Novus Biologicals, Littleton, CO, United States), prior to lysis of exosomes for quantification of cargo proteins via ELISA. In our prior work in a cohort of subjects with clinically overt vascular disease we used Nanosight LM10 instrument (Malvern Instruments, Malvern, United Kingdom) to confirm that the particle we were immunoprecipitating were within range for exosomes (Goetzl et al., 2017) and that particle counts correlated with CD81 concentrations. We used exactly the same technique for the work being reported in this manuscript, and therefore we are making the assumption that the immunoprecipitated particles are also within size range for exosomes, and we normalized to CD81. It should be noted that we performed two rounds of affinity purification with monoclocal antibodies to enhance selectivity, however, we cannot entirely exclude the possibility that our EDE preparation still contains other plasma membrane-derived vesicles within size range of exosomes.

### Enzyme-Linked Immunosorbent Assay Quantification of Proteins

Endothelial-derived exosomes cargo proteins were quantified by ELISA kits for brain-expressed GLUT-1 (glucose transporter 1), NOSTRIN (Nitric Oxide Synthase Traffic Inducer), P-GP (permeability-glycoprotein, or ABCB1), LAT1 (large neutral amino acid transporter), VCAM-1 (Vascular Cell Adhesion Molecule 1), and the tetraspanning exosome marker CD81 (Cusabio; American Research Products, Waltham, MA, United States) according to manufacturer’s protocols. CD81 was used as a surrogate marker of exosome concentration against which each protein was normalized.

### Quantification of Plasma Analytes

Plasma cytokine concentrations were measured by high-performance electrochemiluminescence (HPE) using the multiplex V-PLEX Human Proinflammatory (IL-6, TNFα) and Human Chemokine (MCP-1/CCL2) panels. We selected IL-6 and TNFα to represent global levels of systemic inflammation and MCP-1/CCL2 to measure monocyte activation. The multiplex arrays were analyzed with a MESO QuickPlex SQ 120 imager (MSD, Rockville, MD, United States) and Discovery Workbench v4.0 software. Concentrations were obtained in duplicate per each sample in accordance with the manufacturer’s protocol.

### Statistical Analyses

Statistical analysis was performed by JMP Pro and PRISMA. Significantly skewed variables (all EDE cargo proteins, plasma cytokines, and WMH) were transformed to normalize distributions. For comparison between cases and controls, Chi-squared tests (for categorical variables) and Student’s *t*-tests for continuous measures of age, global cognitive score, and blood pressure were used. Unpaired Student’s *t*-tests, as well as analysis of covariance (ANCOVA) were used to determine biomarkers that significantly differed between cases and controls, adjusting for age. Logistic regression was employed for ROC analyses. Pearson’s correlation was used to analyze the relationships between EDE biomarkers with cognition and gray matter atrophy, correcting for age and total intracranial volume. Where appropriate, we performed a stringent Bonferroni correction for multiple comparisons.

## Results

### Demographics

There were no significant differences with respect to sex and education between groups. Clinical and laboratory-based vascular risk values (i.e., systolic blood pressure, LDL, triglycerides, HgbA1C) were also not significantly different between the groups (*p* > 0.05). Cases were significantly older than controls (*p* = 0.0074) (Table 1). We therefore controlled for age in all our analyses. MMSE did not significantly differ between groups (*p* = 0.7).

**Table 1:** Participant demographics.

	Controls	Cases (cSVD)	*p*-Value
Total number	15	11	–
F (n, %)	6, 40%	5, 45%	–
Age, mean (SEM)	72 (2)	80 (2)	0.0003^∗^
Education, mean (SEM)	18 (0.5)	18 (0.6)	0.9
CDR	0	0	–
MMSE, mean (SEM)	29 (0.2)	29 (0.3)	0.7
Processing speed	21(6)	34 (14)	0.007^∗^

*Age and processing speed are significantly different between groups. Group comparisons performed by ANOVA.*

### Group Differences

**FIGURE 1:**
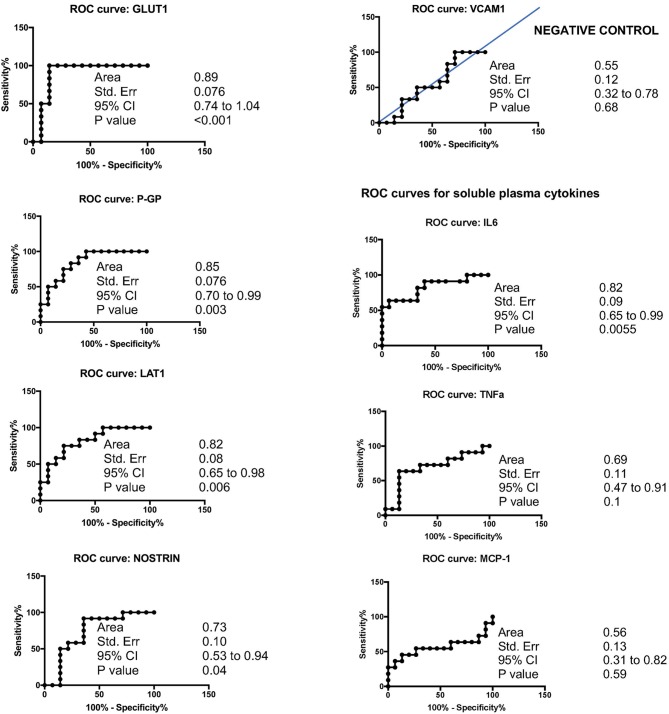
Accuracy of EDE cargo proteins in preclinical cSVD.

Overall, cSVD cases showed significantly higher levels of brain-expressed EDE cargo proteins compared to controls with unpaired Student’s *t*-tests two-tailed *p*-values <0.0001. These differences remained highly significant after controlling for age (ANCOVA *p*-values <0.0001 to 0.0012). VCAM1, which has the lowest reported expression in cerebral vascular endothelial cells, showed no significant difference between the groups with Student’s *t*-tests (*p* = 0.22). Overall, individual markers were associated with areas under the curve (AUC) values ranging 0.7 to 0.9 (Figure 1). Entering a second marker into the discriminant function evaluation did not substantially improve the sensitivity or specificity of the classification (Table 2). The greatest group difference in EDE protein concentrations were, in order of magnitude: GLUT1, P-GP, LAT1, and NOSTRIN. The AUC for the strongest marker (GLUT1) was 0.89 (SE 0.076, 95% CI 0.744–1.042), with optimal diagnostic accuracy cut off >755 pg/ml, associated with a likelihood ratio (LR) of 7 at sensitivity of 100% and specificity of 85%.

**Table 2:** Accuracy of EDE cargo proteins.

	AUC	Std. Error	95% Cl	*p*-Value	Cut off value (pg/mL)	Sensitivity %	Cl 95%	Specificity %	Cl 95%	Likelihood Ratio
GLUT1	0.89	0.076	0.74–1.04	<0.001	>755	100	74–100	86	57–98	7
LAT1	0.82	0.083	0.65–0.98	0.006	>1624	75	43–95	79	50–95	3.5
P-GP	0.85	0.076	0.70–0.99	0.003	>21551	75	43–95	79	50–95	3.5
NOSTRIN	0.73	0.10	0.53–0.94	0.04	>539	92	62–100	64	35–87	2.6
VCAM1	0.55	0.12	0.32–0.78	0.68	>6135	50	21–79	64	35–87	1.4

### Relation With Brain Imaging

**FIGURE 2:**
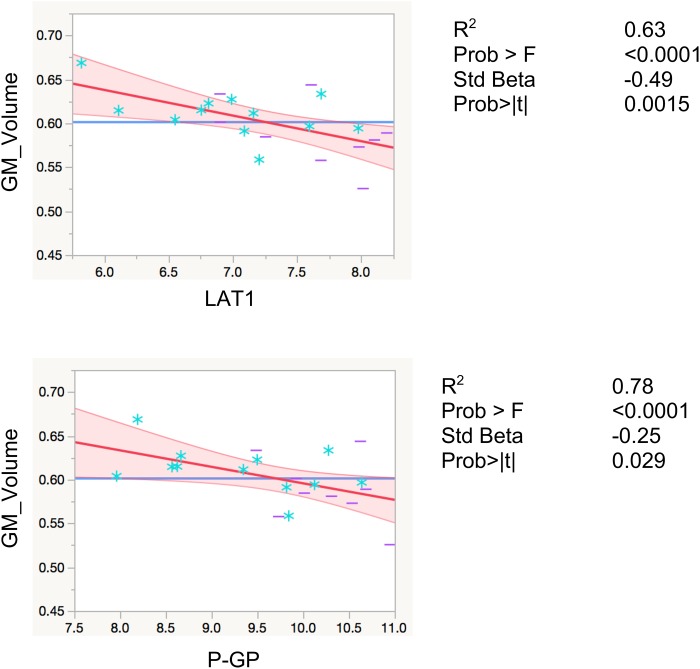
Regression models with global gray matter volume. Associations of EDE cargo proteins with total gray matter volumes. This figure shows leverage residuals plots of global gray matter volumes regressed on normalized EDE cargo biomarkers. Units for gray matter volumes entered into the model are mm^3^. Age in years and total intracranial volumes in mm^3^ were controlled for in all regressions. *R*^2^ values are adjusted for number of predictors in regression model. All values are rounded to two significant digits. GM, gray matter; blue stars, controls; purple dashes, cases.

LAT1 levels were significantly inversely associated with total gray matter volumes, controlling for total intracranial volume and age (*R*^2^ = 0.80, *F* < 0.0001, β = −0.29, *p* = 0.0086). Levels of P-GP were also inversely associated with total gray matter volumes (*R*^2^ = 0.78, *F* < 0.0001, β = −25, *p* = 0.029), however, the association did not reach Bonferroni-adjusted significance level of *p* < 0.01 (Figure 2). The inverse associations for GLUT1 and NOSTRIN, were also in anticipated direction, however, did not reach significance (*p* = 0.23 for GLUT1 and *p* = 0.11 for NOSTRIN).

### Relation With Cognitive Function

**FIGURE 3:**
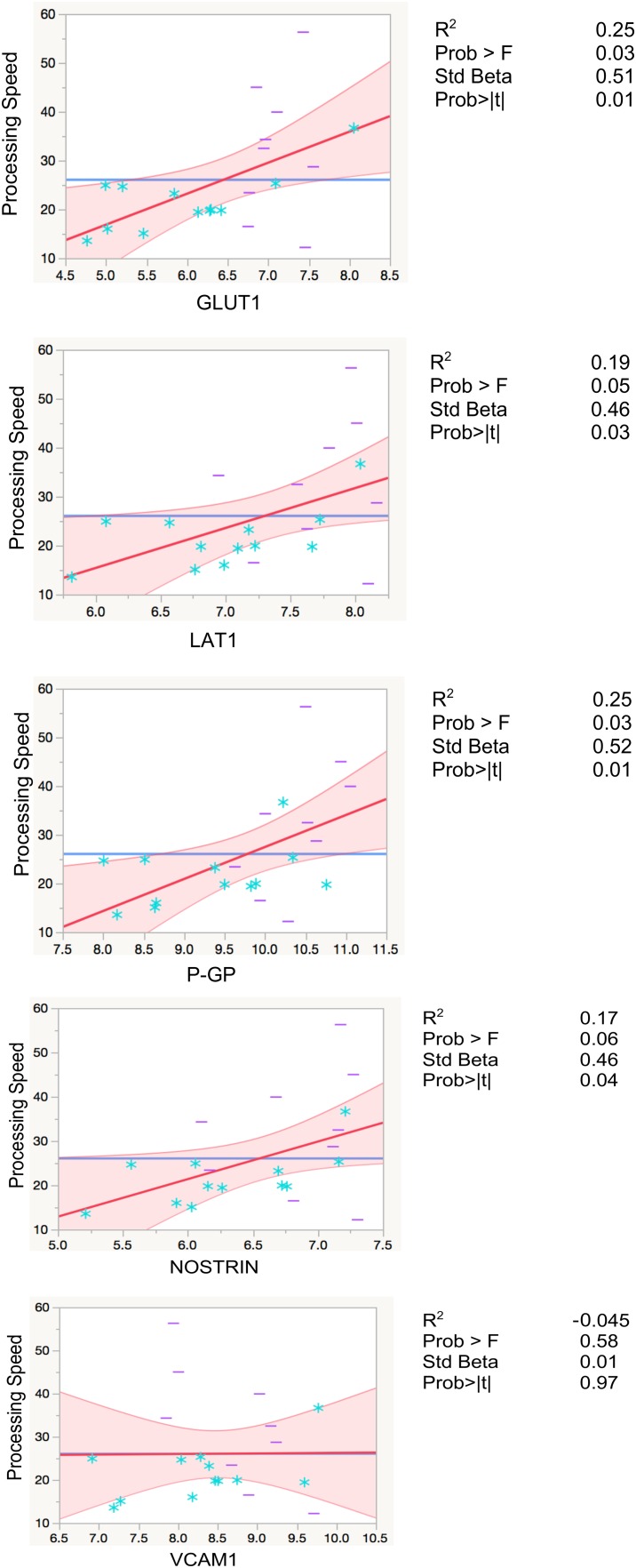
Regression models with processing speed. Leverage residuals plots of normalized levels of EDE cargo biomarkers associated with cognition (processing speed) where units are in seconds. Distributions were normalized via log transformation. All regressions were adjusted for age. *R*^2^ values are adjusted for number of predictors in regression model. All values are rounded to two significant digits. Blue stars: controls; purple dashes: cases.

Higher levels of EDE cargo proteins were associated with lower cognitive function as measured by set-shifting performance speed, controlling for age in all analyses (GLUT1: β = 0.5, *p* = 0.01; LAT1: β = 0.46, *p* = 0.03; P-GP: β = 0.51, *p* = 0.01; NOSTRIN: β = 0.46, *p* = 0.04). GLUT1 and P-GP remained significant after Bonferroni correction for multiple comparisons (Figure 3 and Table 3).

**Table 3:** Results from linear regression models between independent variables of EDE cargo proteins, plasma cytokines, and neuroimaging, with processing speed as dependent variable.

	GLUT1	LAT1	P-GP	NOSTRIN	VCAM1	IL-6	MCP-1	TNFα	WMH	GM
*R*^2^	0.2	0.2	0.3	0.14	−0.05	0.2	0.2	−0.009	−0.006	−0.02
β	0.5	0.46	0.6	0.43	0.07	0.5	0.5	0.2	0.37	0.5
*p*-Value	0.02**^∗^**	0.03**^∗^**	0.009**^∗^**	0.06	0.7	0.02**^∗^**	0.04**^∗^**	0.4	0.3	0.3
Prob >F	0.04	0.06	0.019	0.1	0.6	0.04	0.08	0.4	0.4	0.5

*Models are adjusted for age and in the case of imaging biomarkers for total intracranial volumes. Distributions were normalized by log transformation. Significant associations are starred.*

### Relation With Secreted Plasma Cytokine and Chemokine Markers

Significant positive associations were found between systemic inflammatory cytokine IL-6 and EDE GLUT1 (β = 0.6; *p* = 0.0032), LAT1 (β = 0.7; *p* = 0.001), and P-GP (β = 0.5; *p* = 0.04). Associations between IL-6 and EDE NOSTRIN approached, but did not reach significance (β = 0.4; *p* = 0.056), and VCAM1 (*p* = 0.2) was not significantly associated with IL-6. Although all associations were positive between EDE markers and MCP-1/CCL2, none reached significance (*p* = 0.08–0.1, and for VCAM1 *p* = 0.3). We did not find any associations with TNFα (*p*-value range 0.5–0.9).

## Discussion

In this case-control study we show that levels of EDE proteins selected based on *a priori* associations with endothelial contributions to brain metabolism, BBB function, and inflammatory regulation, were significantly higher in clinically normal subjects with evidence of WMH in comparison to those without WMH.

In light of known associations between inflammation and vascular disease (Desikan et al., 2015; Ridker et al., 2017), we also investigated the association of IL-6, TNFα and MCP-1/CCL2 with normalized levels of EDE cargo proteins. It has been shown that proinflammatory cytokines upregulate GLUT1 in endothelial cells and astrocytes (Adhikari et al., 2011; Winkler et al., 2015; Jais et al., 2016; Ferrer, 2017; Schüler et al., 2018). Consistent with these reported findings, we showed that a state of chronic sterile inflammation represented by IL-6 plasma levels is associated with higher concentrations of brain expressed EDE cargo, namely GLUT1, LAT-1, and P-GP. We did not find an association with TNFα. This may be due to technical issues such as stability of analytes, measurement techniques, or fundamental biological differences between the roles of these inflammatory cytokines. For instance, IL-6 is a known downstream target of IL-1β, consistently increased in patients with NLRP3 inflammasome-mediated conditions, such as cardiovascular disease and associated with clinical outcomes (Ridker et al., 2017).

Associations of EDE proteins with gray matter volumes did not hold for all markers. We found a significant inverse association of LAT-1 with gray matter volumes, with a stringent Bonferroni-corrected significance threshold (*p* < 0.01). Interestingly, the EDE markers with high expression in brain endothelia showed strong significant associations with cognitive function. NOSTRIN’s association with cognitive function reached significance with a higher *p*-value than the other three markers (*p* = 0.04), and VCAM1 (which has the lowest reported levels of expression in brain endothelia), was not associated with cognitive function. As a complex, multifactorial outcome, cognition is the culmination of many converging biological pathways, regulated at numerous levels and impacted by multiple pathologies (Snyder et al., 2015; Bos et al., 2017). The strength of associations we found, despite the relative small size of our cohort, speaks to the signal-to-noise ratio of EDE biomarkers. In addition, our findings provide the first *in vivo* evidence in humans and underscore previously suggested role of endothelial vascular health and BBB integrity for brain structure and function (DeCarli, 2013; Iadecola, 2013; Friedman and Kaufer, 2015; Montagne et al., 2015, 2016).

We did not find associations among GM and WMH volumes with our cognitive variable (Table 3). In light of heterogeneity in age-associated changes in brain structure and function, our study was not powered for the investigation of structural MRI variables with cognition, which have been previously investigated in much larger cohorts (Brickman et al., 2007). The fact that we found strong, significant associations among EDE proteins with cognition speaks to the relative effect sizes of EDE biomarkers in comparison to structural brain imaging markers.

The high discriminant accuracy obtained in our study suggests that EDE molecular cargo, in addition to increased specificity for endotheliopathy, have high sensitivity in clinically normal subjects. Longitudinal studies are needed to determine how well these exosome markers of endotheliopathy predict changes in brain structure and declines in cognition.

The marker used for immunoprecipitation of EDE is not specific to brain EDE. However, our cohort of clinically normal older subjects, selected based on cerebral radiographic criteria of asymptomatic disease, did not have evidence of significant systemic vascular disease. Despite isolation of all EDE, our signal-to-noise was such that we found high effect sizes for proteins with high cerebral endothelial expression levels, namely GLUT1, P-GP, and LAT-1, followed by NOSTRIN, which is ubiquitously expressed in all vascular beds. The marker that is systemic rather than brain expressed, VCAM1, did not show a difference between groups. These findings confirm the utility of EDE biomarkers for study of cerebral processes in absence of significant clinically evident systemic disease.

**FIGURE 4:**
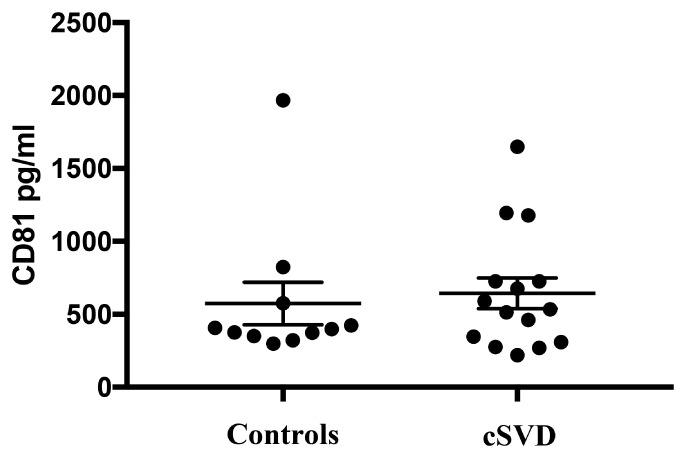
Levels of CD81, the exosomal marker used to normalize EDE cargo are not significantly different between groups (*p* = 0.69).

Given that diseased endothelia are thought to release higher numbers of exosomes (Hromada et al., 2017), we normalized the concentrations of EDE cargo to CD81 (Figure 4), a ubiquitous exosome marker. We can therefore conclude that the noted upregulation of brain-expressed EDE proteins in cases versus controls represents pathophysiological changes within endothelia, even in clinically asymptomatic individuals. Such changes may be instigating and driving the pathological process, or potentially represent a reactive, or even homeostatic response to molecular and cellular changes occurring at the BBB, including within endothelia. Alternatively, diseased endothelia may be sending these proteins to neighboring cells, since exosomes are a known means of communication between cells (Budnik et al., 2016).

The noted increased concentrations of EDE GLUT1 in our subjects with WMH may be reflecting early metabolic changes. The normal adult brain constitutes approximately 2% of the body weight and consumes approximately 20% of glucose in the body. The aerobic metabolism of glucose is the main source of energy in the CNS. The interaction among endothelial cells, astrocytes, and neurons, forming the neurovascular unit, has a central role in coupling energy supply to demand, according to changes in neuronal activity (Iadecola, 2004). Metabolic changes have been reported in vascular disease with and without dementia (Pascual et al., 2010), and associated with burden of WMH and executive function (Haight et al., 2013).

In order to therapeutically target pathways, we need a deeper understanding of molecular differences and dysregulated pathways involved in the plethora of vasculopathies. This study constitutes a step in this direction. In addition to putative metabolic dysregulations discussed above, changes in LAT-1 and P-GP concentrations suggest alterations to the permeability of endothelial cells, and NOSTRIN concentrations reflect putative inflammation and perfusion changes. We selected a negative control, VCAM1, based on the premise that isolated WMH on brain imaging in the absence of systemic signs and symptoms of vascular disease may reflect differential vulnerabilities of organ-specific endothelia to injury. In contrast to high levels of systemic expression, brain expression of VCAM1 seems limited to select venous cerebral endothelial beds (Podtelezhnikov et al., 2014; Vanlandewijck et al., 2018). Therefore, despite group differences in VCAM1 demonstrated in subjects with systemic vascular occlusive disease versus controls (Goetzl et al., 2017), as expected, we did not find differences in levels between participants with evidence of WMH and absence of vascular occlusive disease (e.g., lacunar strokes, angina, or myocardial infarctions) from controls.

Replication of findings in an independent cohort is always advisable, however, based on the selection method, the effect sizes we obtained, the statistical strength of our positive and negative findings, and the existence of a prior study showing high accuracy of these EDE cargo proteins in cohorts with clinical evidence of vascular disease, we believe that our findings are highly likely to be generalizable. Importantly, we undertook several steps to support the validity of our reported findings. First, we included a negative control, VCAM1. Second, the selection of subjects for this study was based on radiographic findings on prior MRI and patient willingness to participate in our prospective study. We performed a consecutive sampling of study participants already enrolled in our longitudinal studies with demographic characteristics that did not differ significantly from the those represented in the parent study.

Exosomes are multivesicular bodies that form from inward budding of intraluminal endosomal vesicles, and contain proteins, lipids, and RNA from their cell of origin (Hromada et al., 2017). They are referred to as “liquid biopsies” in cancer diagnostics, given their ability to reflect the milieu of their cell of origin and are rapidly emerging as promising non-invasive fluid markers of disease (Hafiane and Daskalopoulou, 2018). In this study we show that exosomes can provide powerful tools for non-invasive “biopsies” of disease states in asymptomatic, and likely preclinical stages of cSVD. The ability to separate subjects with high versus low WMH suggests that EDE molecular cargo could provide the means of patient selection in preclinical disease stage at a point when implementation of therapeutics would be optimal. In addition, such biomarkers could also be used for assessment of target engagement, should clinical trials investigate therapeutic interventions aimed at cerebral vascular endotheliopathies.

Biomarkers directly associated with cerebral endotheliopathies offer a much-needed specificity for the study of endothelial contributions to BBB dysfunction and down-stream degenerative changes. In addition, they offer the unique opportunity to quantify contributions of vascular endotheliopathies to the spectrum of neurodegenerative disorders, including Alzheimer’s disease, frontotemporal lobar dementias, and synucleinopathies (Skillbäck et al., 2017). Focus on the study of *in vivo* endothelial molecular changes provides valuable insights into specific molecular pathways with potential improvement of early disease detection and guidance of preventative therapeutic developments. To our knowledge this is the first report of *in vivo* associations between molecular changes in endothelia with WMH, a radiographic finding that is hypothesized to be downstream of cSVD. The findings in our study suggest that exosome-derived molecules may provide *in vivo* insights into the multicellular pathogenesis of cerebral degenerative changes.

## Author Contributions

FME contributed to the project conceptualization, design, data acquisition, statistical analyses, interpretation of results, and manuscript preparation. KBC and AMS contributed to discussions and revision of the manuscript. MRA coordinated the study. EF contributed to brain imaging processing. CD contributed to project discussions, brain imaging processing, and revision of manuscript. MMG and TJF contributed to the statistical advice and verification of analyses. JDH contributed to project discussions, and revision of the manuscript. BLM contributed to project discussions and acquired funding. EJG contributed to critical project discussions, data acquisition, interpretation, and critical revision of the manuscript. JHK contributed to project discussions, critical revision of manuscript, and funding acquisition.

## Conflict of Interest Statement

The authors declare that the research was conducted in the absence of any commercial or financial relationships that could be construed as a potential conflict of interest.
